# Ultrasound contrast-enhanced radiomics model for preoperative prediction of the tumor grade of clear cell renal cell carcinoma: an exploratory study

**DOI:** 10.1186/s12880-024-01317-1

**Published:** 2024-06-06

**Authors:** Yujie Luo, Xiaoling Liu, Yiping Jia, Qin Zhao

**Affiliations:** 1 Department of Ultrasound, West China School of Public Health and West China Fourth Hospital, Sichuan University, No. 18, Section 3, Renmin South Road, Wuhou District, Chengdu, Sichuan 610041 China; 2 Department of Ultrasound, Nanchong Central Hospital (Nanchong Clinical Research Center), The Second Clinical Medical College, Nanchong Central Hospital, North Sichuan Medical College (University), Nanchong, Sichuan 637000 China

**Keywords:** Renal clear cell carcinoma, WHO/ISUP, Nuclear classification, Contrast-enhanced ultrasound. Radiomics

## Abstract

**Background:**

This study aims to explore machine learning(ML) methods for non-invasive assessment of WHO/ISUP nuclear grading in clear cell renal cell carcinoma(ccRCC) using contrast-enhanced ultrasound(CEUS) radiomics.

**Methods:**

This retrospective study included 122 patients diagnosed as ccRCC after surgical resection. They were divided into a training set (*n* = 86) and a testing set(*n* = 36). CEUS radiographic features were extracted from CEUS images, and XGBoost ML models (US, CP, and MP model) with independent features at different phases were established. Multivariate regression analysis was performed on the characteristics of different radiomics phases to determine the indicators used for developing the prediction model of the combined CEUS model and establishing the XGBoost model. The training set was used to train the above four kinds of radiomics models, which were then tested in the testing set. Radiologists evaluated tumor characteristics, established a CEUS reading model, and compared the diagnostic efficacy of CEUS reading model with independent characteristics and combined CEUS model prediction models.

**Results:**

The combined CEUS radiomics model demonstrated the best performance in the training set, with an area under the curve (AUC) of 0.84, accuracy of 0.779, sensitivity of 0.717, specificity of 0.879, positive predictive value (PPV) of 0.905, and negative predictive value (NPV) of0.659. In the testing set, the AUC was 0.811, with an accuracy of 0.784, sensitivity of 0.783, specificity of 0.786, PPV of 0.857, and NPV of 0.688.

**Conclusions:**

The radiomics model based on CEUS exhibits high accuracy in non-invasive prediction of ccRCC. This model can be utilized for non-invasive detection of WHO/ISUP nuclear grading of ccRCC and can serve as an effective tool to assist clinical decision-making processes.

**Supplementary Information:**

The online version contains supplementary material available at 10.1186/s12880-024-01317-1.

## Introduction

Clear cell renal cell carcinoma(ccRCC) is the most common solid lesion of the kidneys and accounts for approximately 90% of all renal malignancies [1]. Tumor nuclear grading is a well-known prognostic factor for ccRCC and is considered an independent predictor of cancer-specific survival [2, 3]. Currently, the widely accepted ccRCC classification system is the World Health Organization/International Society for Urology and Pathology (WHO/ISUP) standard established in 2016 [4]; according to the WHO/ISUP standard, ccRCC is classified into four grades from I-IV based on the increase in nucleolar protrusion and the presence of extreme nuclear pleomorphism and tumor giant cell and sarcomatoid and rhabdomyoid differentiation. It can also be simplified into a binary classification standard, dividing nuclear grades I–II into lower grades and nuclear grades III–IV into higher grades. High-grade ccRCC is invasive, prone to metastasis, and has a poor prognosis. Predicting tumor grade in advance can help determine appropriate treatment strategies [5, 6]. Percutaneous pathological biopsy, which often causes bleeding, is a commonly used method for the preoperative grading of ccRCC. Moreover, owing to the heterogeneity of ccRCC, there are some inconsistencies between biopsy and resected samples in the WHO/ISUP grading system [7]. Therefore, there is an urgent need for a non-invasive and effective method to determine the histological grading of small ccRCCs.

Contrast-enhanced ultrasonography (CEUS) shows real-time tissue perfusion with excellent spatial and temporal resolution [8]. It has been widely used in kidney examinations because of its unique advantages, such as the absence of radiation, repeatability, and convenience [9–11]. Zhao et al. suggested that both CEUS and DCE-MRI can quantify tumor perfusion, blood volume, and capillary grade permeability, demonstrating the hemodynamic characteristics of tumors and effectively distinguishing ccRCC from non-ccRCC. Therefore, CEUS can serve as an alternative to DCE-MRI, and the cost-effectiveness of MRI is relatively poor compared with ultrasound [12]. Recent studies used CEUS to evaluate the degree of ccRCC differentiation, and blood flow perfusion was found to be closely related to the degree of differentiation [13, 14]. Although CEUS can intuitively reveal the blood flow perfusion of renal tumors, texture information is present in images that the naked eye cannot observe, and its diagnostic efficacy is still limited. In recent years, ultrasound radiomics has been increasingly applied to image analysis. Ultrasonics uses computer programming techniques to extract quantitative textural features and to detect high-dimensional images. Combining these biomarkers with machine learning (ML) technologies can effectively identify complex tissue changes [15, 16]. However, there is no research on ultrasound radiomics ML models based on ultrasound images to predict the degree of RCC differentiation. This study aimed to establish a radiomics ML model based on renal cancer CEUS images to predict the degree of differentiation before surgery.

## Methods

### Patients and datasets

This research was approved by the Institutional Review Committee of the West China School of Public Health and West China Fourth Hospital, and the requirement for informed consent was waived. CEUS images of completely resected ccRCCs from December 2017 to January 2024 at the West China School of Public Health, West China Fourth Hospital, and Nanchong Central Hospital were used in this study. A total of 122 ccRCC patients were obtained from the image databases of the two hospitals to form a training and testing set in a 7:3 ratio. The inclusion criteria were: (1) ccRCC patients who underwent partial or radical nephrectomy. (2) Patients who underwent CEUS examination within two weeks before surgery, (3) patients with complete clinical data, and (4) no previous renal surgery or other treatment performed on suspected ccRCC lesions. The exclusion criteria were as follows: (1) patients who underwent anticancer therapy (such as radiotherapy, chemotherapy, and ablation) before CEUS examination; (2) patients with a history of both kidney tumors and other types of tumors; and (3) patients with CEUS image loss or poor image quality.

### CEUS image acquisition

The US instruments used to acquire the images in this study included IU22 and EPIQ7 (Philips, Amsterdam, the Netherlands) and GE LOGIQ E9 (General Electric Co., USA). First, grayscale ultrasound was used to examine the upper abdomen, and the scanning sound window, depth, gain, dynamic range, mechanical index, output power, and focal area of the mass were adjusted to obtain the optimal CEUS image. Ultrasound contrast agents (SonoVue; Bracco, Italy) were used, and 1.5 ml of the contrast agent was injected through the elbow vein. Subsequently, the cells were washed with 5 ml of physiological saline solution. The timer began counting after the injection of the contrast agent. A low mechanical index (MI < 0.1) was used for the CEUS examination. According to the guidelines, CEUS examination is divided into a renal cortical phase (CP) 15–30 s after UCA administration with clinical enhancement seen and a renal medullary phase(MP), where both cortical enhancement and medullary enhancement occur 25s–4 min after UCA administration [8]. Gray-scale images of the patient’s most significant area, cortical tumor image, and medullary tumor image for analysis.

### Pathological evaluation

Two pathologists evaluated all cases by observing hematoxylin and eosin (HE)-stained sections under a microscope. All cases were classified according to the standards of the 2016 WHO/ISUP grading system [4]. Divide ccRCC tumors into low-grade (grades I and II) and high-grade (grades III and IV) groups according to the 2016 WHO/ISUP grading system [4]. If there was a difference in opinion, another senior pathologist was invited to participate in the discussion and reach a consensus.

### CEUS analysis

Ultrasound examination was retrospectively evaluated by two expert radiologists (engaged in CEUS work for approximately 9 and 5 years) on ultrasound contrast images of all patients unaware of the pathological and clinical results. Two reviewers independently evaluated the following imaging features of each ccRCC: (a) size; (b) echogenicity(Hyper/Iso/Hypo); (c) shape(irregular/regular); (d) margin (unclear/clear); (e) CEUS enhance speed (when the enhancement intensity of cortical tumors is greater than or equal to the surrounding renal parenchyma, it is classified as “fast”; otherwise, it is considered as “slow”); (f) washout (comparison of enhancement intensity and renal cortical echo intensity in medullary stage tumors); (g) the pseudocapsule(yes/no); (h) necrosis(yes/no). If there was a difference in the CEUS results of the patient, the two people were evaluated after discussion.

### Image segmentation and ultrasound radiomics feature extraction

**Fig. 1:**
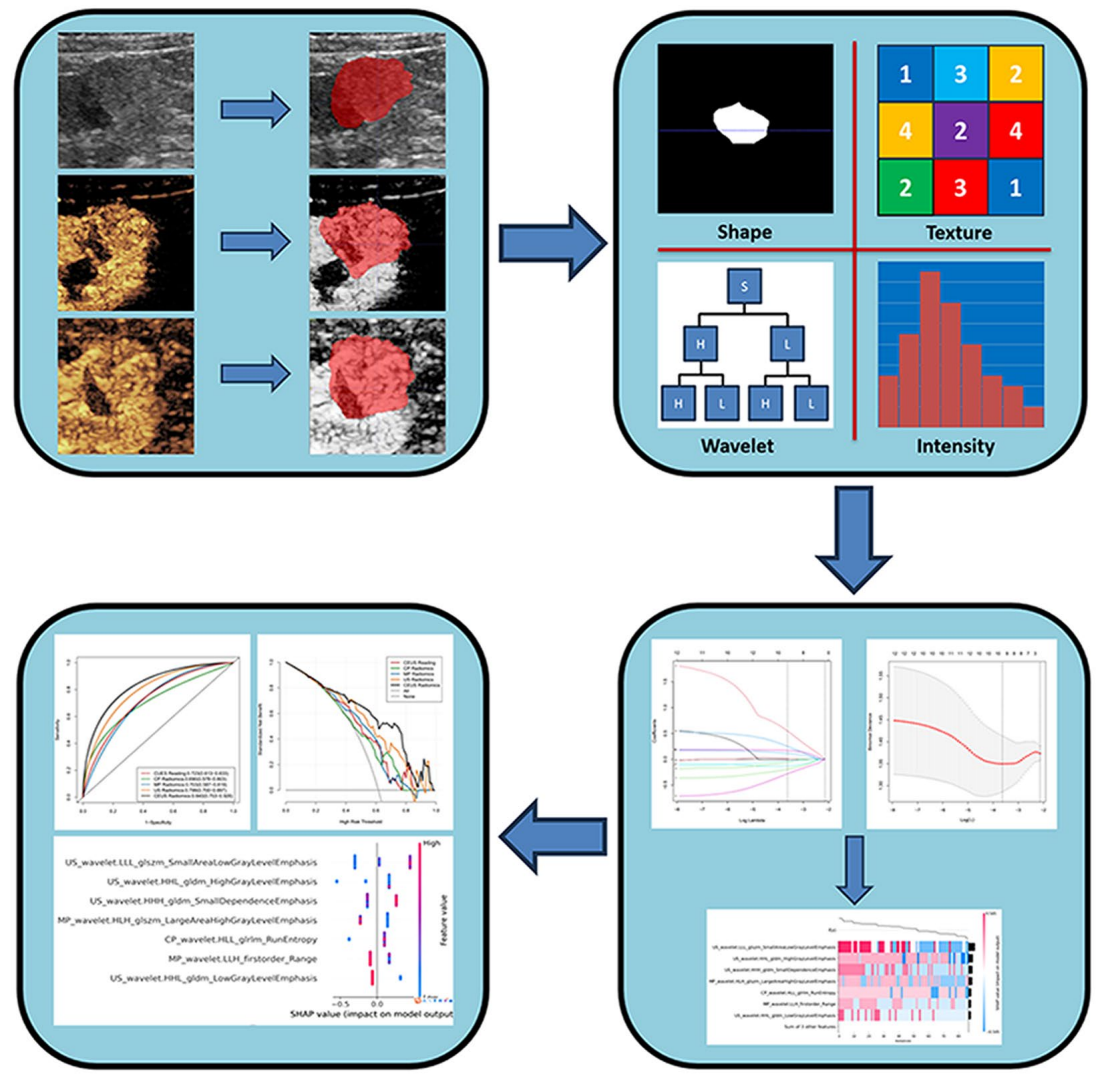
The ultrasound radiomics flow chart of the study

We took the following steps in the preprocessing process. First, we standardized the image grayscale to ensure consistency in the grayscale distribution in the image. Next, we adopted image-denoising techniques to reduce the possible interference of noise during feature extraction. In addition, we resampled the images using a linear interpolation algorithm to obtain a standardized voxel spacing of 1 × 1 × 1 mm (x, y, z). We used ITK software(3.8.0, help://www.itksnap.org/pmwiki/pmwiki.php? n=Downloads.SNAP3)for manual segmentation, a widely used tool in medical image processing. The manual segmentation process is not limited to conventional two-dimensional ultrasound images but includes enhanced ultrasound cortical phase images and ultrasound medullary phase images. The area of interest refers to the entire lesion area. During the delineation process, special attention is paid to ensure that the area of interest fully covers the lesion to obtain accurate lesion boundaries. In addition, to reduce errors, all manual segmentations were independently performed by two experienced radiologists (with 10 and 7 years of experience in abdominal ultrasound diagnosis), and discussions and negotiations were conducted as needed to reach a consensus on the segmentation results. We used intra- and inter-class correlation coefficients (ICC) to evaluate feature stability. Specifically, we randomly selected renal ultrasound images from 50 patients and had two radiologists calibrate their respective regions of interest (ROIs). Subsequently, Radiologist 1 repeated the same steps two weeks later and extracted the imaging omics features. We evaluated the consistency and stability of the feature extraction by calculating the intra- and inter-group correlation coefficients of these three sets of features. To ensure the reliability of the results, we set a threshold for ICC values greater than 0.75, indicating that these features have good consistency and stability and are suitable for subsequent quantitative analysis. The remaining images were independently ROI-segmented by radiologist 1, and only features with good correlation and stability were retained for subsequent analyses. This strategy is adopted to ensure the analysis’s accuracy and reliability while avoiding potential errors caused by segmentation inconsistency or feature instability. Using PyRadiomics (version 3.0.1, https://github.com/AIM-Harvard/pyradiomics.)extracting radiomics features from ultrasound images. Implemented numerous engineering-coding feature algorithms. The steps for screening ultrasound omics features and constructing ultrasound omics models are shown in Fig. 1: Firstly, the features with ICC > 0.75 in the training set were retained. Second, a single-factor rank-sum test was used to screen for statistically significant feature differences between the training set’s high- and low-grade groups. Subsequently, Pearson’s correlation analysis was performed on the remaining radiomics features to remove highly correlated features. Finally, the LASSO algorithm is used to select the optimal features, and the Xgboost algorithm is used to establish a radiomics model.

To overcome the “black box” nature of ML models, the SHAP method [17] explains each variable’s impact on the optimal performance model. The SHAP method is based on alliance game theory and calculates the SHAP value, which evaluates each variable’s marginal contribution to the model’s final prediction. Several cases were proposed to reveal how the best model generated each prediction. In addition, the SHAP values for each variable in all patients were summarized and averaged to obtain a queue view for global interpretation.

### Statistical analysis

All statistical analyses were conducted using SPSS (version 25.0; IBM Corp., Armonk, NY, USA) and Python 2.7 (Python Software Foundation, Beaverton, OR, USA). Quantitative data with normal distribution is represented as standard deviation. Categorical variables are expressed as numbers and percentages. The chi-square test, two independent sample t-tests, and Mann-Whitney U-test were used for univariate analysis. Logistic regression analysis performed univariate and multivariate analyses of the clinical parameters. Statistical significance was set at *p* < 0.05. significant.

The receiver operating characteristic(ROC) curve, area under the curve (AUC), and decision curve analysis(DCA) were used to evaluate the predictive performance, calibration ability, and clinical practicality of the models. Other distinguishing indicators included accuracy, sensitivity, specificity, positive predictive value(PPV), and negative predictive value(NPV).

## Results

**Fig. 2:**
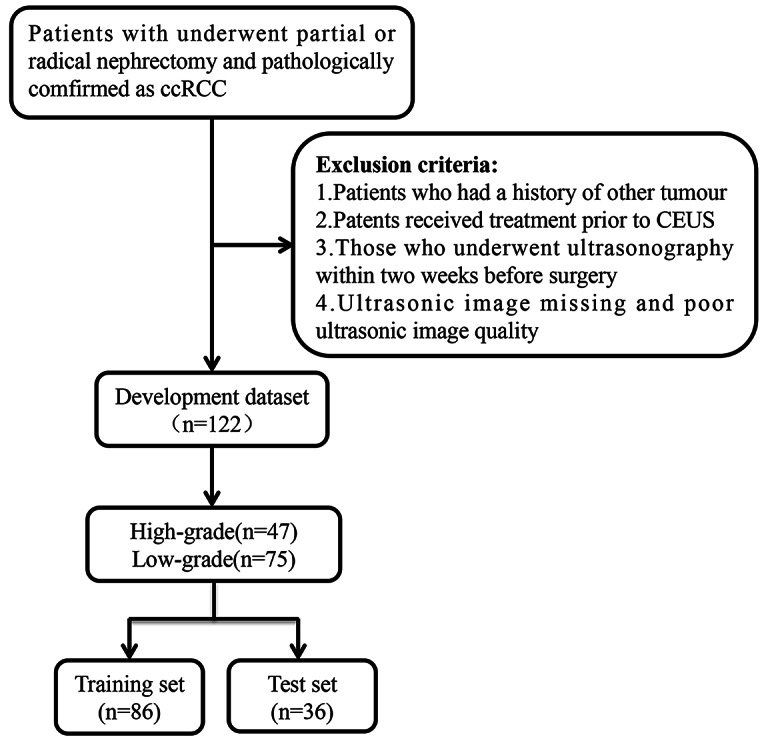
Patint flowchart

The basic clinical characteristics of the patients are shown in Table 1. Among the 122 ccRCC patients included in the study, there were 84 males and 38 females, with a mean age of 57.6 ± 13.1 years (ranging from 21 to 81 years), Among them, 75 cases (61.5%) were classified as low grade, while 47 cases(38.5%) were classified as high grade. After randomly assigning patients at a ratio of 7:3, they were divided into a training group (*n* = 86) and a testing group (*n* = 36). The patient selection process is illustrated in Fig. 2.

**Table 1:** Clinical factors in the training and testing group

	Training	Testing
Sex(Male/Female)	56/30	28/8
Age (Years)	57.1 ± 13.1	58.9 ± 13.2
Size(mm)	48.1 ± 26.4	46.2.1 ± 22.1
Shape(Irregular / Regular)	23/63	8/28
Margin (Unclear / Clear)	60/26	27/9
Echogenicity(Hyper /Iso / Hypo)	31/27/28	19/6/11
Washin (Fast/Slow)	76/10	33/3
Washout (Yes/No)	46/40	20/16
Necrosis(Yes/No)	49/37	24/36
Pseudocapsule(Yes/No)	42/44	25/11

### CEUS reading models

In the univariate analysis, significant statistical differences were observed between several CEUS parameters and the ccRCC grade, specifically “size”(*P* = 0.003), “washout”(*P* = 0.017) and “necrosis”(P = 0.02). Subsequent multiple factor analysis identified “size” (P = 0.035) and “washout” (P = 0.032) as independent predictive factors. A CEUS model was then established, and in the training set, the AUC, accuracy, sensitivity, and specificity of CEUS predicting the WHO/ISUP nuclear grading of ccRCC were 0.723, 0.698, 0.606, and 0.755, respectively. In the testing set, the AUC, accuracy, sensitivity, and specificity were 0.695, 0.722, 0.929, and 0.591, respectively.

### Feature extraction, selection, and construction of radiomics features

**Fig. 3:**
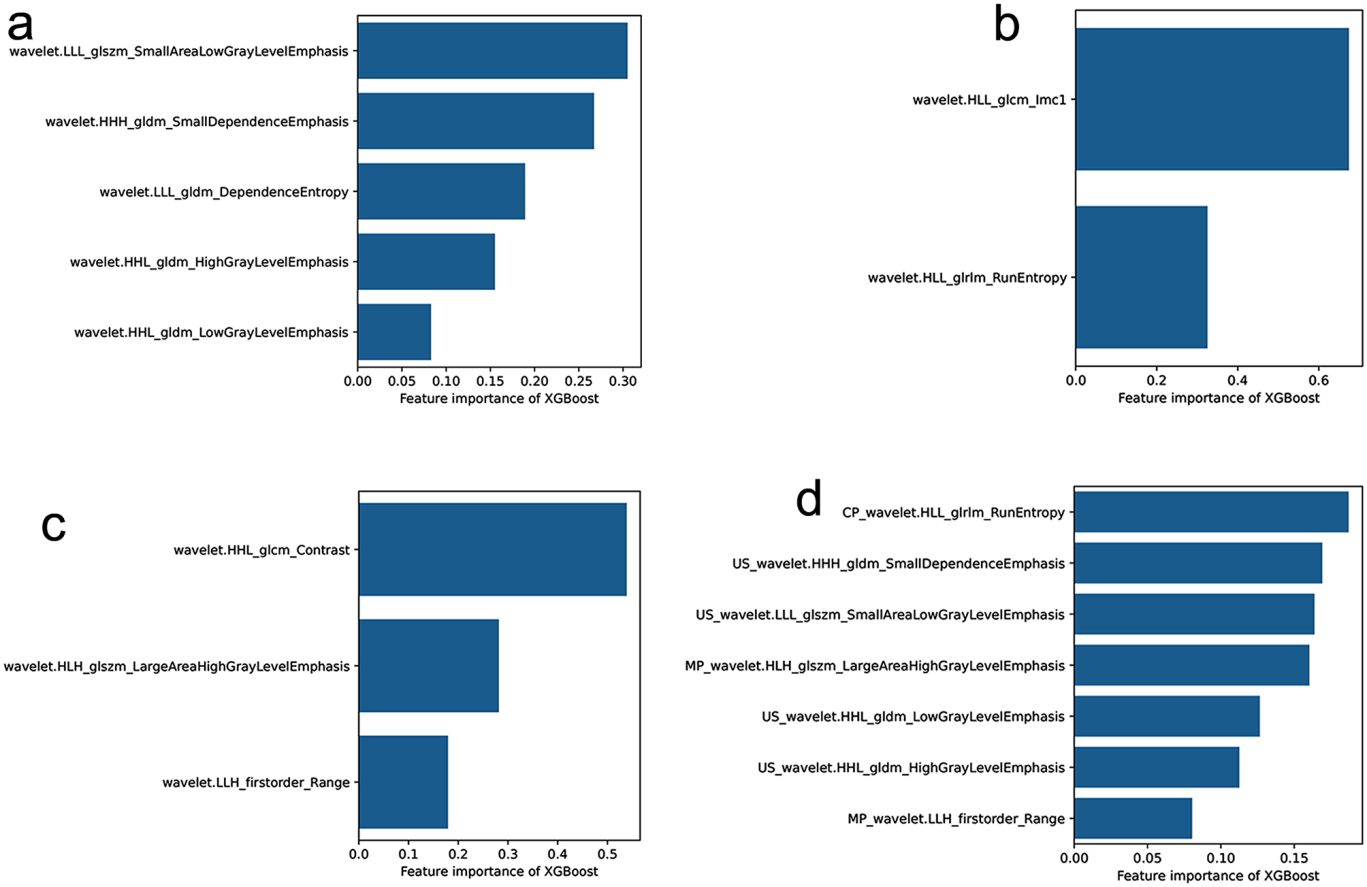
The radiomic features of the four radiomic model.A.the US radiomic features B. The CP radiomic features C. The MP radiomic features D. The CEUS radiomic features

A total of 873 radiomics features were extracted from gray scale US images. After conducting Inter-group and Intra-group Correlation Coefficient(ICC), along with univariate correlation analysis, it was found that there were significant differences in 80 features between the two groups. These features were sequentially subjected to univariate rank sum test and Pearson correlation analysis, followed by LASSO dimensionality reduction. Finally, a total of 5 optimal features were obtained, as shown in Fig. 3A.Similarly, a total of 873 radiomics features were extracted from CEUS images of CP. After ICC, as well as univariate correlation analysis, it was found that there were significant differences in 52 features between the two groups. These features were sequentially subjected to univariate rank sum test and Pearson correlation analysis, followed by LASSO dimensionality reduction, to obtain a total of 2 optimal CP radiomics features, as shown in Fig. 3B. A total of 873 radiomics features were extracted from the CEUS images of MP. After ICC, as well as univariate correlation analysis, 48 features were found to have significant differences between the two groups. These features were sequentially subjected to univariate rank sum test and Pearson correlation analysis, and LASSO dimensionality reduction was performed. Finally, a total of 5 optimal MP radiomics features were obtained, as shown in Fig. 3C. The Xgboost algorithm was used to establish individual US, CP and MP models for the selected optimal radiological characteristics of each group. Logistic regression analysis was carried out on the radiomic features extracted from CP, MP and US ultrasound images, and a total of 10 optimal radiomic features were finally obtained, as shown in Fig. 3D; Table 2. Xgboost algorithm is also used to build the combined CEUS radiomics ML model .

**Table 2:** Coefficients of Selected features in the ultrasound radiomics model

The Features of CEUS Radiomics	Coefficient	OR
CP_wavelet.HLL_glrlm_RunEntropy	-0.2785	0.756918
MP_wavelet.LLH_firstorder_Range	-0.19001	0.826952
MP_wavelet.LHL_ngtdm_Strength	-0.12568	0.881894
MP_wavelet.LHH_glszm_ZoneEntropy	-0.05502	0.94647
MP_wavelet.HLH_glszm_LargeAreaHighGrayLevelEmphasis	-0.0331	0.967438
US_wavelet.HHL_gldm_HighGrayLevelEmphasis	0.006546	1.006568
US_wavelet.HHL_gldm_LowGrayLevelEmphasis	0.113886	1.120624
US_wavelet.HHH_gldm_SmallDependenceEmphasis	0.171702	1.187324
US_wavelet.LLL_gldm_DependenceEntropy	0.2514	1.285825
US_wavelet.LLL_glszm_SmallAreaLowGrayLevelEmphasis	0.550671	1.734416

**Fig. 4:**
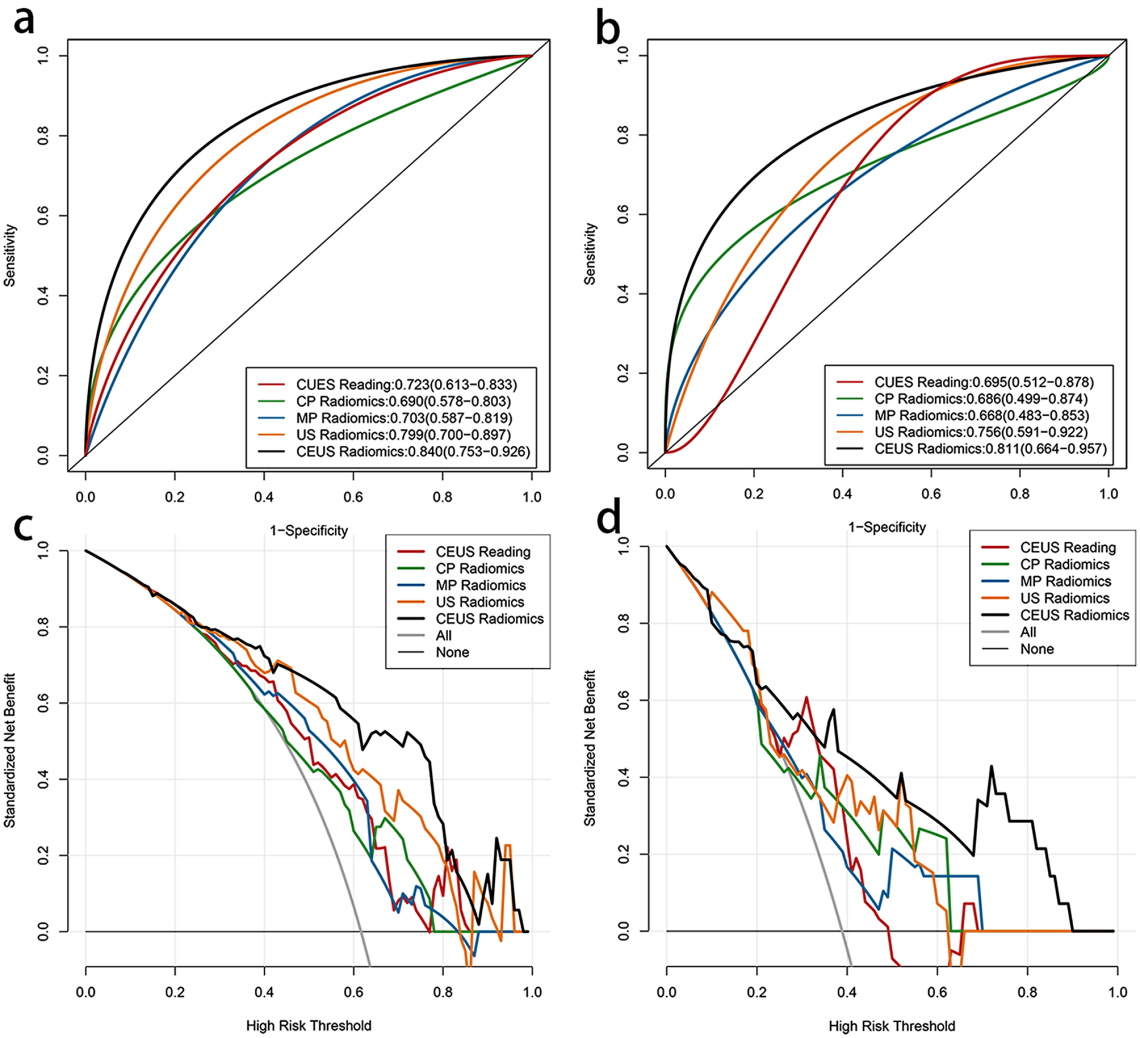
A and C show the ROC and DCA curves of the five models in the training group.B and D represent the ROC and DCA curves of five models in the test group

**Fig. 5:**
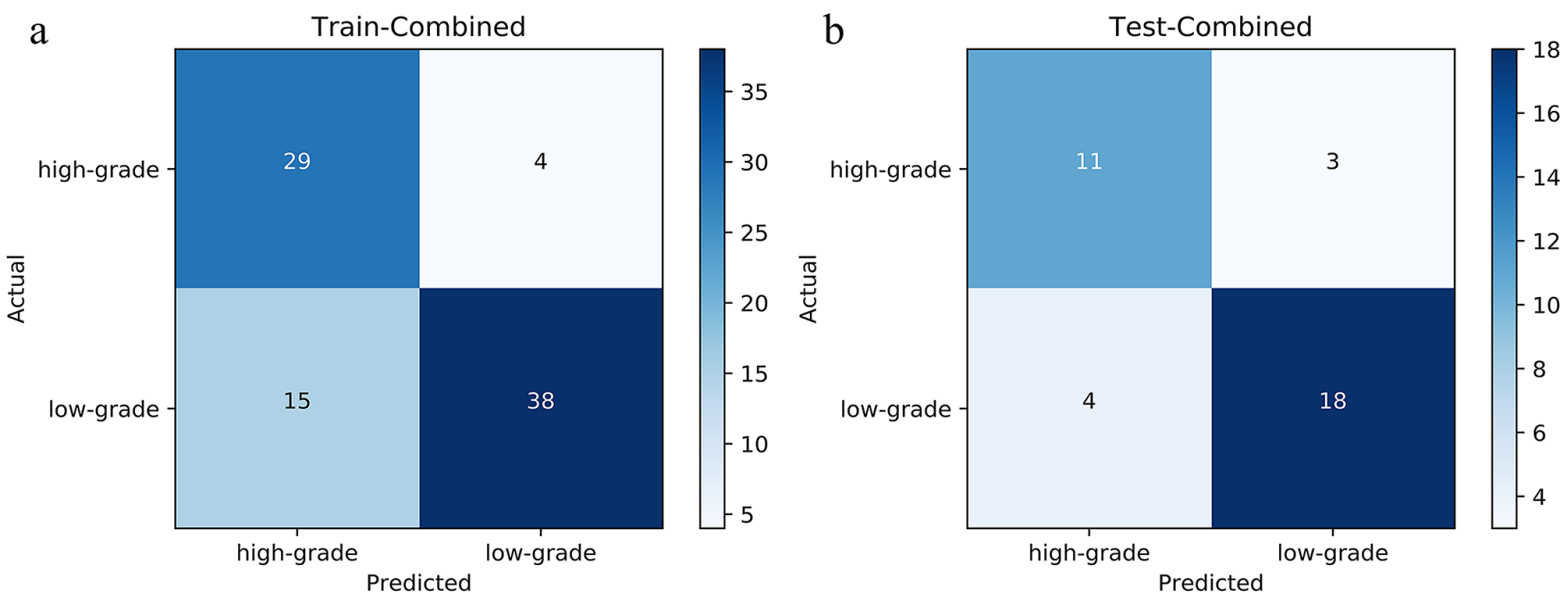
The confusion matrix of the combined models. (A) The confusion matrix of the combined models in the training set. (B) The confusion matrix of the combined models in the testing set. True and predicted subtype classifications are shown on the y- and x-axes, respectively. The blue gradient color represents the model accuracy for detecting each subtype. The darker the blue color, the better the model performance

The results, as depicted in Fig. 4; Table 3, reveal that in the models of the training set and the testing set, the AU*C* value of the combined CEUS radiomics model surpasses that of the the individual US, CP and MP radiomics model, as well as the CEUS reading model. The AUC of the training set is 0.84, with an accuracy rate of 0.779, sensitivity of 0.717, and specificity of 0.879. The AUC of the testing set is 0.811, with an accuracy rate of 0.784, sensitivity of 0.783, and specificity of 0.786. Furthermore, DCA demonstrates that both in the training and the testing sets, the combined CEUS radiomics model yield a higher overall net income than the other four models. The confusion matrix of the combined models of the training set and the testing set was showed in Fig. 5.

**Table 3:** Performance of four radiomics models and clinical model in the training and the testing group

Model	AUC (95%CI)	ACC	SEN	SPE	PPV	NPV	Precision	F1
Training group								
CEUS Radiomics	0.84 (0.753–0.926)	77.9%	71.7%	87.9%	90.5%	65.9%	90.5%	80%
US Radiomics	0.799 (0.7-0.897)	79.1%	86.8%	66.7%	80.7%	75.9%	80.7%	83.6%
CP Radiomics	0.69 (0.578–0.803)	65.1%	56.6%	78.8%	81.1%	53.1%	81.1%	66.7%
MP Radiomics	70.3 (0.587–0.819)	70.9%	79.2%	57.6%	75%	63.3%	75%	77.1%
CEUS Reading	0.723 (0.613–0.833)	69.8%	60.6%	75.5%	60.6%	75.5%	75.5%	75.5%
**Testing group**								
CEUS Radiomics	0.811 (0.664–0.957)	78.4%	78.3%	78.6%	85.7%	68.8%	85.7%	83.7
US Radiomics	0.756 (0.591–0.922)	78.4%	87%	64.3%	80%	75%	79.2%	82.6%
CP Radiomics	0.686 (0.499–0.874)	70.3%	73.9%	64.3%	77.3%	60%	77.3%	77.3%
MP Radiomics	0.668 (0.483–0.853)	67.6%	82.6%	42.9%	70.4%	60%	70.4%	77.6%
CEUS Reading	0.695 (0.512–0.878)	72.2%	92.9%	59.1%	59.1%	92.9%	93.9%	72.2%

AUC = area under the curve; ACC = accuracy; SEN = sensitivity; SPE = specificity; PPV = positive predictive value, NPV = negative predictive value

**Fig. 6:**
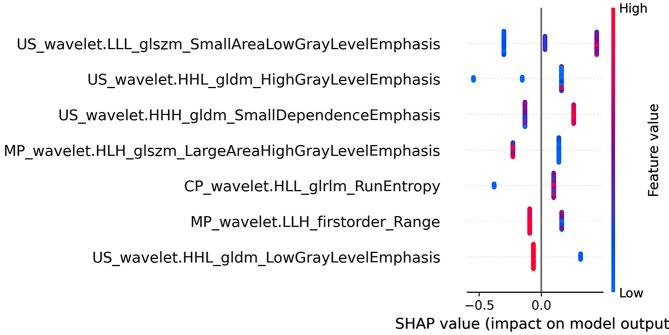
shows the global interpretation of the SHAP Beeswarm diagram

**Fig. 7:**
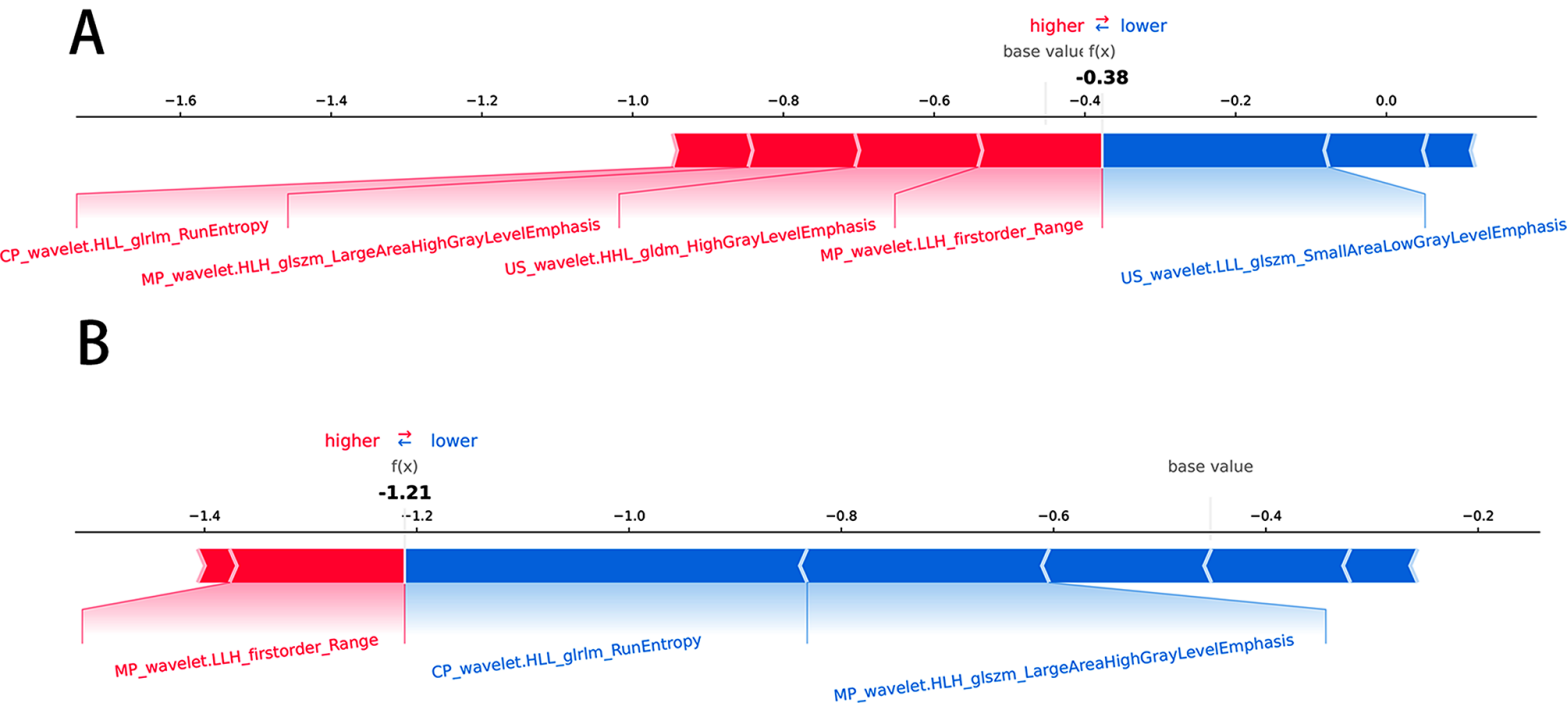
Individual prediction of the model using the SHAP plot method. A.Patient A has high-grade ccRCC B. Patient B has low-grade ccRCC

As shown in Fig. 6, the importance of features was ranked using a Beesworm plot. In addition, two patients (patients A and B) were randomly selected to explain the individual prediction of the model using the SHAP plot method. As shown in the figure, the graph generated by SHAP (Fig. 7) shows that the red color indicates that the variable increases the chance of the model predicting patients as adverse outcomes, while the blue color indicates that the variable reduces the chance of the model predicting patients as adverse outcomes. The impact of each feature on the model’s classification output can be seen through the scale values on the X-axis.

## Discussion

In this study, a radiomics ML model was developed to predict ccRCC grades using CEUS. The model produced satisfactory predictions for the training and testing sets, with the AUC of ROC being 0.84 and 0.811, respectively. In addition, the diagnostic performance of the model was superior to that of the CEUS prediction model based on radiologists’ readings. The results demonstrate the feasibility of using a CEUS radiomics model to predict the WHO/ISUP grading of ccRCC. This is the first study to use the CEUS radiomics ML model to predict the WHO/ISUP grading of ccRCC.

Curative surgery is acceptable for high-grade ccRCC, whereas minimally invasive techniques such as nephron-sparing surgery and ablation are more feasible for low-grade ccRCC [18]. Precise preoperative histological grading is crucial for monitoring patient condition and developing personalized follow-up treatment strategies. Recently, Huang collected CEUS qualitative and quantitative features from 69 ccRCC patients confirmed by surgical pathology and found a statistical difference between the blood flow perfusion of lesion contrast-enhanced ultrasound and the WHO/ISUP grading of ccRCC. The higher the degree of enhancement, the richer the blood vessels, the more active the cell growth, and the more prominent the nucleolus [13]. In our study, multiple-factor analysis was conducted by radiologists to evaluate the CEUS features of tumors, and it was found that tumor size and washout were independent predictive factors for preoperative ccRCC grading. Consistent with previous studies, washout may be caused by an incomplete neovascular wall leading to increased permeability, disrupted vascular beds, abnormal channels, and the formation of a large number of arteriovenous fistulas; the rich internal lymphatic network and rapid reflux of the contrast agent lead to rapid fading of the contrast agent [14].

Furthermore, we established a predictive model and obtained results with a training group ROC of 0.723 and a testing group ROC of 0.695; however, the results did not meet clinical needs. Radiomics can reveal subtle differences in the intensity distribution of medical images that the human eye cannot easily recognize, and the signal distribution pattern reflects the heterogeneity of tumors [19–21]. There is a correlation between textural features and pathological changes caused by diseases. Radiomics can extract and quantify perceptual ranges of high-throughput imaging biomarkers in humans. Radiomic indicators can serve as helpful ccRCC [22–24].

Grey-level normalization is required when radiomic features are extracted from US images to ensure the accuracy and reliability of the results [25]. After feature selection, we selected ten ultrasonic radiomics features to evaluate ccRCC and the ccRCC.The CEUS radiomics model was established by collecting tumor CEUS images of the US, CP, and MP. The results showed that the ultrasound imaging radiomics model has a strong processing ability for high-throughput data, and the ultrasound radiomics model based on CEUS can effectively predict ccRCC with better results than the prediction model based on radiologist readings. Although previous studies have suggested that MRI-based radiomics and machine learning methods can predict Fuhrman grading [26], CEUS is cheaper and more convenient and can better reflect the blood flow perfusion of tumors. To overcome the “black box” nature of ML models, SHAP is used to visualize the importance of model features. The SHAP value allocates the probability of the model output to each feature. This can help understand how much each feature contributes to the prediction results, making the ML model’s predictions more transparent and interpretable. It can be seen that although the US provides an essential contribution to the model’s performance, the CEUS performance in the cortical and medullary stages of tumors is also an indispensable contribution to the non-invasive diagnosis of ccRCC in the model.

This retrospective study had some limitations. First, it was a retrospective study with a selection bias, and the results depended on the composition of limited-sized data. Second, because of the retrospective extraction of images from the three stages of the tumor for analysis, static images may lead to the loss of helpful information compared with dynamic images. Third, no external validation was performed. This limits the assessment of the clinical value of the proposed model because there is no information on the degree of challenge posed by the cases or the added accuracy provided by the model. To verify the feasibility of our radiomics column chart, future carefully designed prospective longitudinal cohort studies must be conducted in a larger patient population and at multiple centers.

In summary, it has been demonstrated that ML models based on radiomics can accurately predict the WHO/ISUP grading of ccRCC through contrast-enhanced ultrasound images. With further validation in more populations in the future, the model has enormous potential and can be used as an essential decision-support tool in clinical applications.

### Electronic supplementary material

Below is the link to the electronic supplementary material.

Supplementary Material 1

Supplementary Material 2

Supplementary Material 3

Supplementary Material 4

## Data Availability

The datasets used and/or analysed during the current study are available from the corresponding author on reasonable request.

## Abbreviations

ML: Machine learning
ccRCC: Clear cell renal cell carcinoma
CEUS: Contrast-enhanced ultrasound
SHAP: SHAPPLEY Additive exPlanations
CP: Cortical phase
MP: Medullary phase

## References

[1] Ljungberg B, Albiges L, Abu-Ghanem Y, Bedke J, Capitanio U, Dabestani S, Fernández-Pello S, Giles RH, Hofmann F, Hora M (2022). European Association of Urology Guidelines on Renal Cell Carcinoma: the 2022 Update. Eur Urol.

[2] Browning L, Colling R, Verrill C (2021). WHO/ISUP grading of clear cell renal cell carcinoma and papillary renal cell carcinoma; validation of grading on the digital pathology platform and perspectives on reproducibility of grade. Diagn Pathol.

[3] Delahunt B, Cheville JC, Martignoni G, Humphrey PA, Magi-Galluzzi C, McKenney J, Egevad L, Algaba F, Moch H, Grignon DJ (2013). The International Society of Urological Pathology (ISUP) grading system for renal cell carcinoma and other prognostic parameters. Am J Surg Pathol.

[4] Moch H, Cubilla AL, Humphrey PA, Reuter VE, Ulbright TM (2016). The 2016 WHO classification of Tumours of the urinary system and male genital organs-Part A: renal, Penile, and testicular tumours. Eur Urol.

[5] Delahunt B, Eble JN, Egevad L, Samaratunga H (2019). Grading of renal cell carcinoma. Histopathology.

[6] Zhao Y, Wu C, Li W, Chen X, Li Z, Liao X, Cui Y, Zhao G, Liu M, Fu Z (2021). 2-[(18)F]FDG PET/CT parameters associated with WHO/ISUP grade in clear cell renal cell carcinoma. Eur J Nucl Med Mol Imaging.

[7] Perrino CM, Cramer HM, Chen S, Idrees MT, Wu HH (2018). World Health Organization (WHO)/International Society of Urological Pathology (ISUP) grading in fine-needle aspiration biopsies of renal masses. Diagn Cytopathol.

[8] Sidhu PS, Cantisani V, Dietrich CF, Gilja OH, Saftoiu A, Bartels E, Bertolotto M, Calliada F, Clevert DA, Cosgrove D (2018). The EFSUMB guidelines and recommendations for the clinical practice of contrast-enhanced Ultrasound (CEUS) in non-hepatic applications: Update 2017 (Long Version). Ultraschall Med.

[9] Pan KH, Jian L, Chen WJ, Nikzad AA, Kong FQ, Bin X, Wang YL, Chen M (2020). Diagnostic performance of contrast-enhanced Ultrasound in Renal Cancer: a Meta-analysis. Front Oncol.

[10] Huang X, Wang N, Liu L, Zhu J, Wang Z, Wang T, Nie F (2023). Pre-operative prediction of invasiveness in renal cell carcinoma: the role of conventional ultrasound and contrast-enhanced Ultrasound. Ultrasound Med Biol.

[11] Stock K, Kübler H, Maurer T, Slotta-Huspenina J, Holzapfel K (2018). [CEUS-diagnosis of solid renal tumors]. Radiologe.

[12] Zhao P, Zhu J, Wang L, Li N, Zhang X, Li J, Luo Y, Li Q (2023). Comparative diagnostic performance of contrast-enhanced ultrasound and dynamic contrast-enhanced magnetic resonance imaging for differentiating clear cell and non-clear cell renal cell carcinoma. Eur Radiol.

[13] Huang X, Nie F, Zhu J, Liu L, Wang N (2023). Diagnostic value of contrast-enhanced Ultrasound features for WHO/ISUP Grading in Renal Cell Carcinoma. J Ultrasound Med.

[14] Fan X, Fu F, Liang R, Xue E, Zhang H, Zhu Y, Ye Q. Associations between contrast-enhanced ultrasound features and WHO/ISUP grade of clear cell renal cell carcinoma: a retrospective study. Int Urol Nephrol 2023. 10.1007/s11255-023-03774-z 37670195

[15] Mu W, Schabath MB, Gillies RJ (2022). Images are data: challenges and opportunities in the clinical translation of Radiomics. Cancer Res.

[16] Qin X, Xia L, Hu X, Xiao W, Huaming X, Xisheng X, Zhang C (2023). A novel clinical-radiomic nomogram for the crescent status in IgA nephropathy. Front Endocrinol.

[17] Lundberg SM, Lee S-I. A unified approach to interpreting model predictions. Adv Neural Inf Process Syst 2017, 30.

[18] Escudier B, Porta C, Schmidinger M, Rioux-Leclercq N, Bex A, Khoo V, Gruenvald V, Horwich A (2016). Renal cell carcinoma: ESMO Clinical Practice guidelines for diagnosis, treatment and follow-up. Ann Oncol.

[19] Liu Z, Wang S, Dong D, Wei J, Fang C, Zhou X, Sun K, Li L, Li B, Wang M (2019). The applications of Radiomics in Precision diagnosis and treatment of Oncology: opportunities and challenges. Theranostics.

[20] Qin X, Xia L, Zhu C, Hu X, Xiao W, Xie X, Zhang C (2023). Noninvasive evaluation of Lupus Nephritis Activity using a Radiomics Machine Learning Model based on Ultrasound. J Inflamm Res.

[21] Bera K, Braman N, Gupta A, Velcheti V, Madabhushi A (2022). Predicting cancer outcomes with radiomics and artificial intelligence in radiology. Nat Reviews Clin Oncol.

[22] Raman AG, Fisher D, Yap F, Oberai A, Duddalwar VA (2024). Radiomics and Artificial Intelligence: renal cell carcinoma. Urol Clin North Am.

[23] Gao Y, Wang X, Zhao X, Zhu C, Li C, Li J, Wu X (2023). Multiphase CT radiomics nomogram for preoperatively predicting the WHO/ISUP nuclear grade of small (< 4 cm) clear cell renal cell carcinoma. BMC Cancer.

[24] Gutiérrez Hidalgo B, Gómez Rivas J, de la Parra I, Marugán MJ, Serrano Á, Hermida Gutiérrez JF, Barrera J, Moreno-Sierra J. The Use of Radiomic Tools in Renal Mass Characterization. *Diagnostics (Basel)* 2023, 13(17). 10.3390/diagnostics13172743 PMC10487148 37685281

[25] Stanzione A, Cuocolo R, Ugga L, Verde F, Romeo V, Brunetti A, Maurea S. Oncologic Imaging and Radiomics: A Walkthrough Review of Methodological Challenges. *Cancers* 2022, 14(19). 10.3390/cancers14194871 PMC9562166 36230793

[26] Stanzione A, Ricciardi C, Cuocolo R, Romeo V, Petrone J, Sarnataro M, Mainenti PP, Improta G, De Rosa F, Insabato L (2020). MRI radiomics for the prediction of Fuhrman Grade in Clear Cell Renal Cell Carcinoma: a machine learning exploratory study. J Digit Imaging.

