# Supplementary material for: Ultrasound contrast-enhanced radiomics model for preoperative prediction of the tumor grade of clear cell renal cell carcinoma: an exploratory study
Source: BMC Med Imaging. 2024 Jun 6;24:135. doi: 10.1186/s12880-024-01317-1 (PMC11155131; doi:10.1186/s12880-024-01317-1)
Supplement: Supplementary file 2 — Supplementary Material 2 [file 12880_2024_1317_MOESM2_ESM.pdf]

```
import pandas as pd
import os
import SimpleITK as sitk
from radiomics.featureextractor import RadiomicsFeatureExtractor
basePath = 'data/featureExtraction'
folders = os.listdir(basePath)
print(folders)
df = pd.DataFrame()
for folder in folders:
    files = os.listdir(os.path.join(basePath,folder))
    for file in files:
        if file.endswith('.nrrd'):
            imageFile = os.path.join(basePath,folder,file)
        if file.endswith('.nrrd'):
            maskFile = os.path.join(basePath,folder,file)
        settings['binWidth'] = 25
        settings['resampledPixelSpacing'] = [1, 1, 1]
        settings['interpolator'] = sitk.sitkBSpline
        settings['normalize'] = True
        settings['normalizeScale'] = 100
        extractor = featureextractor.RadiomicsFeatureExtractor(**settings, geometryTolerance=1e-5)
        extractor.enableImageTypes(Original={}, Square={}, Wavelet={})
        featureVector = extractor.execute(imageFile, maskFile)
        df_new = pd.DataFrame.from_dict([featureVector.values()])
        df_new.columns = featureVector.keys()
        df = pd.concat([df, df_new])

df.to_excel(os.path.join(basePath,'training160-209.xlsx'))
```
