# Supplementary material for: Ultrasound contrast-enhanced radiomics model for preoperative prediction of the tumor grade of clear cell renal cell carcinoma: an exploratory study
Source: BMC Med Imaging. 2024 Jun 6;24:135. doi: 10.1186/s12880-024-01317-1 (PMC11155131; doi:10.1186/s12880-024-01317-1)
Supplement: Supplementary file 3 — Supplementary Material 3 [file 12880_2024_1317_MOESM3_ESM.pdf]

CLEAR Checklist v1.0

Note: Use the checklist in conjunction with the main text for clarification of all items.
Yes, details provided; No, details not provided; n/e, not essential; n/a, not applicable; Page, page number

Section	No.	Item	Yes	No	n/a	Page
**Title**						
	1	Relevant title, specifying the radiomic methodology	✓			1
**Abstract**						
	2	Structured summary with relevant information	✓			2
**Keywords**						
	3	Relevant keywords for radiomics	✓			3
**Introduction**						
	4	Scientific or clinical background	✓			4-5
	5	Rationale for using a radiomic approach	✓			4-5
	6	Study objective(s)	✓			5
**Method**						
Study design	7	Adherence to guidelines or checklists (e.g., CLEAR checklist)	✓			6
	8	Ethical details (e.g., approval, consent, data protection)	✓			6
	9	Sample size calculation	✓			6
	10	Study nature (e.g., retrospective, prospective)	✓			6
	11	Eligibility criteria	✓			6
	12	Flowchart for technical pipeline	✓			6
Data	13	Data source (e.g., private, public)	✓			6
	14	Data overlap			✓	
	15	Data split methodology	✓			6
	16	Imaging protocol (i.e., image acquisition and processing)	✓			6-7
	17	Definition of non-radiomic predictor variables	✓			6,11
	18	Definition of the reference standard (i.e., outcome variable)	✓			7
Segmentation	19	Segmentation strategy	✓			8
	20	Details of operators performing segmentation	✓			8
Pre-processing	21	Image pre-processing details	✓			8
	22	Resampling method and its parameters	✓			8-9

	23	Discretization method and its parameters	☑	☐	☐	8-9
	24	Image types (e.g., original, filtered, transformed)	☑	☐	☐	8
Feature extraction	25	Feature extraction method	☑	☐	☐	8-9
	26	Feature classes	☑	☐	☐	8-9
	27	Number of features	☑	☐	☐	8-9
	28	Default configuration statement for remaining parameters	☑	☐	☐	8-9
Data preparation	29	Handling of missing data	☐	☐	☑	
	30	Details of class imbalance	☐	☐	☑	
	31	Details of segmentation reliability analysis	☑	☐	☐	9
	32	Feature scaling details (e.g., normalization, standardization)	☑	☐	☐	9
	33	Dimension reduction details	☑	☐	☐	9
Modeling	34	Algorithm details	☑	☐	☐	9
	35	Training and tuning details	☑	☐	☐	9
	36	Handling of confounders	☑	☐	☐	9
	37	Model selection strategy	☑	☐	☐	9
Evaluation	38	Testing technique (e.g., internal, external)	☑	☐	☐	9
	39	Performance metrics and rationale for choosing	☑	☐	☐	10
	40	Uncertainty evaluation and measures (e.g., confidence intervals)	☑	☐	☐	10
	41	Statistical performance comparison (e.g., DeLong's test)	☑	☐	☐	10
	42	Comparison with non-radiomic and combined methods	☑	☐	☐	10
	43	Interpretability and explainability methods	☑	☐	☐	10
**Results**						
	44	Baseline demographic and clinical characteristics	☑	☐	☐	10
	45	Flowchart for eligibility criteria	☑	☐	☐	6
	46	Feature statistics (e.g., reproducibility, feature selection)	☑	☐	☐	11
	47	Model performance evaluation	☑	☐	☐	11-13
	48	Comparison with non-radiomic and combined approaches	☑	☐	☐	12
**Discussion**						
	49	Overview of important findings	☑	☐	☐	13-15
	50	Previous works with differences from the current study	☑	☐	☐	13-15
	51	Practical implications	☑	☐	☐	13-15
	52	Strengths and limitations (e.g., bias and generalizability	☑	☐	☐	15

		issues)				
**Open Science**						
Data availability	53	Sharing images along with segmentation data [n/e]	☐	☐	☑	
	54	Sharing radiomic feature data	☑	☐	☐	9
Code availability	55	Sharing pre-processing scripts or settings	☐	☐	☑	
	56	Sharing source code for modeling	☐	☐	☑	
Model availability	57	Sharing final model files	☑	☐	☐	11-13
	58	Sharing a ready-to-use system [n/e]	☑	☐	☐	11-13

Kocak B, Baessler B, Bakas S, Cuocolo R, Fedorov A, Maier-Hein L, Mercaldo N, Müller H, Orlhac F, Pinto Dos Santos D, Stanzione A, Ugga L, Zwanenburg A. CheckList for EvaluAtion of Radiomics research (CLEAR): a step-by-step reporting guideline for authors and reviewers endorsed by ESR and EuSoMII. Insights Imaging. 2023 May 4;14(1):75. doi: 10.1186/s13244-023-01415-8
