# Supplementary material for: Ultrasound contrast-enhanced radiomics model for preoperative prediction of the tumor grade of clear cell renal cell carcinoma: an exploratory study
Source: BMC Med Imaging. 2024 Jun 6;24:135. doi: 10.1186/s12880-024-01317-1 (PMC11155131; doi:10.1186/s12880-024-01317-1)
Supplement: Supplementary file 4 — Supplementary Material 4 [file 12880_2024_1317_MOESM4_ESM.pdf]

**Supplementary legends**

**Supplementary 1** Radiomic Quality Score, RQS

**Supplementary 2** Clinical Decision-making and Evaluation of Algorithmic Radiomics Studies (CLEAR) Checklist

**Supplementary 3** Pyradiomics Settings File
